# Ultrasonographic measurement of the optic nerve sheath diameter to detect intracranial hypertension: an observational study

**DOI:** 10.1186/s13089-022-00304-3

**Published:** 2023-02-02

**Authors:** Christian Daniel Yic, Julio Pontet, Mauricio Mercado, Matias Muñoz, Alberto Biestro

**Affiliations:** 1 Department of Critical Care Medicine, Hospital de Clínicas, Asociación Española Primera en Salud, Guillermo Arrospide, 5338 Montevideo, Uruguay; 2 Department of Critical Care Medicine, Hospital Pasteur, Montevideo, Uruguay; 3 Comisión Honoraria para la salud Cardiovascular, Montevideo, Uruguay

**Keywords:** Ocular ultrasound, Elevated intracranial pressure, Optic nerve sheath, Acute brain injury

## Abstract

**Objectives:**

To evaluate the ultrasonographic measurement of optic nerve sheath diameter (ONSD) as a predictor of intracranial hypertension as compared to the invasive measurement of intracranial pressure (ICP).

**Design:**

Cross-sectional observational study.

**Setting:**

Intensive Care Unit (ICU) of two tertiary university hospitals in Montevideo, Uruguay.

**Patients:**

We included 56 adult patients, over 18 years of age, who required sedation, mechanical ventilation, and invasive ICP monitoring as a result of a severe acute neurologic injury (traumatic or non-traumatic) and had a Glascow Coma Score (GCS) equal to or less than 8 on admission to the ICU.

**Interventions:**

Ultrasonographic measurement of ONSD to detect intracranial hypertension.

**Measurements and main results:**

In our study, a logistic regression model was performed in which it was observed that the variable ONSD is statistically significant with a *p* value of 0.00803 (< 0.05). This model estimates and predicts the probability that a patient will have an ICP greater than 20 mmHg. From the analysis of the cut-off points, it is observed that a value of 5.7 mm of ONSD maximizes the sensitivity (92.9%) of the method (a greater number of individuals with ICP > 20 mmHg are correctly identified).

**Conclusions:**

In sedated neurocritical patients, with structural Acute Brain Injury, the ONSD measurement correlates with the invasive measurement of ICP. It was observed that with ONSD values less than 5.7 mm, the probability of being in the presence of ICP above 20 mmHg is very low, while for ONSD values greater than 5.7 mm, said probability clearly increases.

## Introduction

The main complication associated with cerebral acute brain injury (ABI) is elevated intracranial pressure (ICP), as it is associated with high morbidity and mortality. Intracranial hypertension is defined as a sustained ICP greater than 20 mm Hg. [1]. In these patients, multimodal neurological monitoring has positioned itself as a fundamental tool, both in emergency departments and in intensive care units (ICU), with a tendency, in recent years, to be minimally invasive. So much so that, in the care of these patients, the detection of increased ICP remains crucial, since it is associated with a worse prognosis [2, 3]. Thus, its early and timely diagnosis together with the implementation of adequate therapeutic measures are essential to ensure better patient outcomes [4]. An integral part of the management of these patients is the implementation of multimodal neurological monitoring, which is based on a series of invasive and non-invasive procedures, each of which has different characteristics and indications. They serve to evaluate the different components that ensure neurological integrity and function, highlighting the measurement of ICP, cerebral perfusion pressure (CPP), cerebral blood flow (CBF), metabolism, oxygen consumption, temperature, and electrical activity, among others [5].

Invasive intracranial devices for monitoring ICP, especially the intraventricular catheter and the intraparenchymal sensor, are considered the gold standard. However, intraventricular catheter is associated with serious complications, among which intracranial hemorrhage (9.2%), ventriculitis (12.2%), and catheter infection (38.6%) [6]. In addition, this type of monitoring is contraindicated in patients with thrombocytopenia or severe coagulopathies. Ultrasound evaluation of the optic nerve sheath diameter (ONSD) has emerged in recent years as a useful non-invasive tool for estimating ICP or detecting intracranial hypertension. It is a safe technique that can be conducted at the bedside, in real time, and is reproducible, relatively low cost, and does not carry the risks of radiation.

Several studies have described this technique but none have validated its accuracy in comparison to the standard invasive measurement of ICP [7].

Recently, Aspide et al. studied the feasibility of using color Doppler to measure the ONSD as compared to 2D ultrasonography, having magnetic resonance imaging as the gold standard, and found that ONSD assessments using color Doppler yielded lower and less scattered measurements compared to 2D ultrasonography [8].

The present study incorporates a regression model from which the probability of finding elevated ICP based on the ONSD measurement can be determined. To date, all studies have been based solely on finding a cut-off point for the ONSD to detect patients with ICP greater than 20 mmHg. None of these studies have included a model that improves the detection of patients with ICP greater than 20 mmHg.

## Objective

To evaluate the ultrasonographic measurement of ONSD as a predictor of intracranial hypertension as compared to the invasive measurement ICP.

## Methods

### Study design and study population

This was a prospective, observational study following the Helsinki Declaration and approved by the institutional review board. Patients older than 18 years who were admitted to the ICU of the Hospital de Clínicas and Asociación Española Primera en Salud, Montevideo, Uruguay, from February 1, 2016 to March 31, 2018 were included.

The exclusion criteria were patients with previous specific traumatic or structural ophthalmological pathology.

Upon admission to the ICU, consent was obtained from patients’ family members. Disease severity was determined following the Glasgow Coma Scale. Electronic medical records were reviewed to obtain demographics (age, gender, race), vital signs (heart rate, respiratory rate, blood pressure, temperature), and hemodynamic parameters (if available).

All patients were sedated with midazolam and fentanyl administered intravenously, according to the treating physician's discretion. Following the decision to place an ICP monitoring device (by the attending intensive care physician and neurosurgeon), the patients were enrolled in this study.

ICP was measured directly through a subdural or ventricular screw or catheter inserted by a neurosurgeon. Intracranial hypertension was considered present when the patient had ICP above 20 mmHg for a period of time greater than 5 min.

The operators measuring the ONSD with ultrasonography were blinded to invasive measurements of ICP.

### Ocular ultrasound

Ocular ultrasound was performed by physicians trained in this technique and according to pre-established protocols [9–13]. On each patient, measurements were performed by a single operator.

The learning curve for optic nerve ultrasound appears to be rapid. In the case of an experienced sonographer, it has been described in the literature that 10 measurements with three abnormal examinations are sufficient to develop technical proficiency. In the case of less experienced operators, up to 25 examinations may be necessary [14, 15].

Retrobulbar ONSD measurement has become possible thanks to the introduction of high-frequency transducers and ultrasound (US) units with a spatial resolution of less than 0.4 mm [16]. According to current protocols, high-frequency linear probes (> 7.5 MHz) should be used to visualize and evaluate the Optic Nerve sheath [13, 17].

All measurements used were carried out with the use of portable, cart-based US equipment, with a linear transducer, at a frequency of 7.5–15 MHz.

The ultrasound presets were established via the "small parts" mode. This setting produces a default field of 4–5 cm depth.

Following FDA regulations for the mechanical index and thermal index for ophthalmic US, we used values less than 0.23 for the mechanical index (MI) and less than 1.0 for the thermal index (TI). Although we used the small parts preset, we lowered the mechanical index below 0.23 by varying the acoustic power [13].

For all patients, the ultrasound beam was focused on the retrobulbar area and the gain was adjusted to obtain the optimal contrast between the optic nerve and the periorbital fat.

The patients were evaluated in the supine position, with the head elevated at 35º. The transducer was placed on the upper eyelid with the patient’s eyes closed, until a hypoechoic line with clearly defined margins posterior to the eyeball was observed.

The probe was always placed gently with the eyelids closed, never on the cornea or sclera, to avoid abrasions or other injury.

**Fig. 1:**
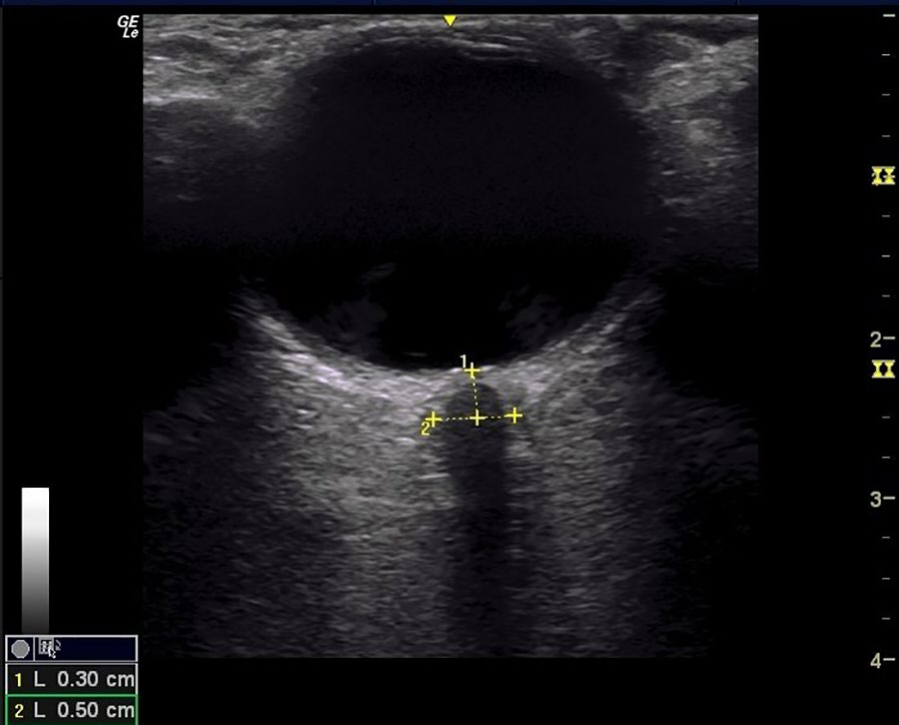
ONSD ultrasound. The ONSD is measured 3 mm behind the retina, from which point a transverse line is drawn from the inner edge to the inner edge of both vertical hypoechoic lines (inner edge of the dura mater)

The ONSD is measured 3 mm behind the retina (Fig. 1), from which point a transverse line is drawn from the inner edge to the inner edge of both vertical hypoechoic lines (inner edge of the dura mater). This has come to be considered the point at which the maximum widening of the ONSD occurs due to the effect of increased ICP [9]. The optic disc is seen as a hyperechoic line at the posterior pole of the eyeball. The normal ultrasound appearance of the optic nerve is considered from the center to the peripheral: hypoechoic nerve fibers closely surrounded by the pia mater with echogenic appearance; the subarachnoid space appears anechogenic or hypoechoic and is surrounded by a hyperechoic dura mater and periorbital fat [7, 13, 18].

**Fig. 2:**
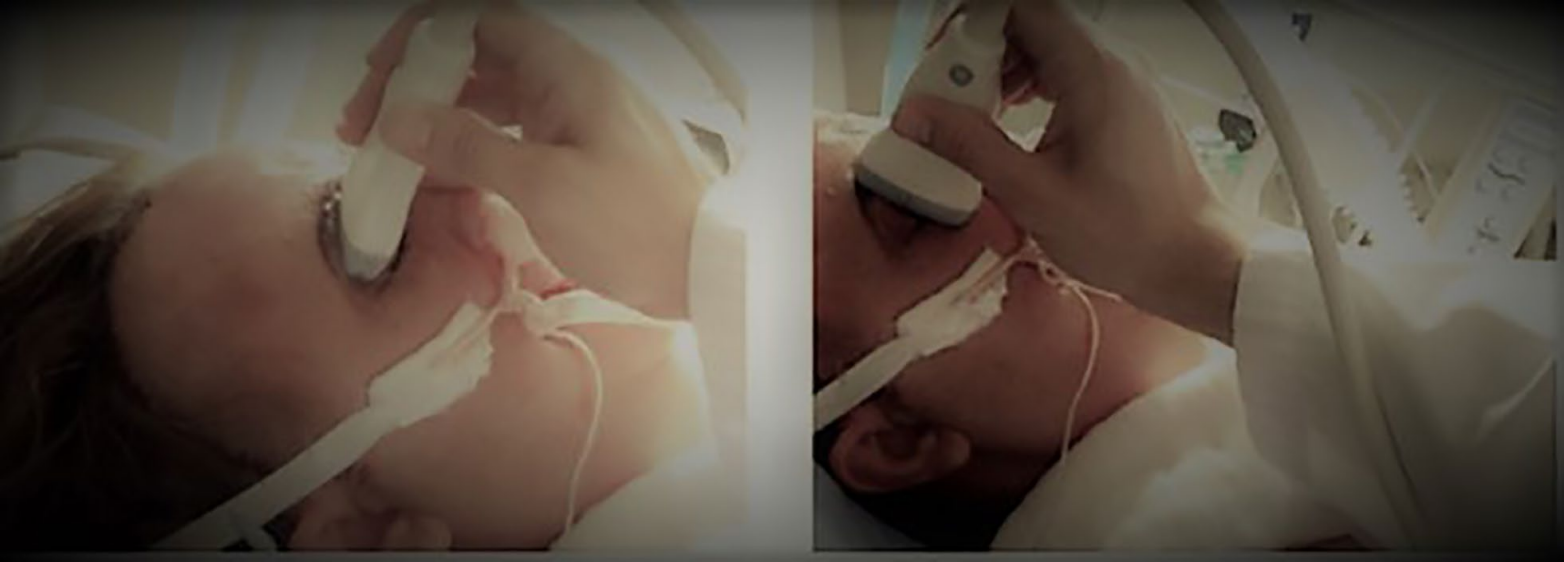
On the left, transducer with transversal orientation for ONSD measurement. To the sagittal flat right with the transducer in the longitudinal direction

For each optic nerve two measurements were made—one in the sagittal plane and the other in the transverse plane—by rotating the probe clockwise (Fig. 2). The mean value obtained for both eyes was retained as the final ONSD value.

In accordance with the Independence assumption, a single ONSD measurement was taken for each patient. The measurements were performed in the ICU after insertion of invasive ICP monitoring.

In all cases, the ICP reading was collected at the end of the ocular ultrasound in order to avoid bias in the measurement.

### Statistical analysis

For the statistical analysis of our data, tables and numerical summaries were made, such as the mean, standard deviation (SD), and range, of the different variables. Using logistic regression models, the relationship between possible predictors and the dichotomous variable ICP > 20 mmHg was studied. Using a simple linear regression model, the relationship between the continuous variables ICP and ONSD was analyzed. Likewise, diagnostic graphs of the model were made.

The ROC curve was graphed and the cut-off points for intracranial hypertension were analyzed by measuring the ONSD. Using bootstrap (non-parametric method) the confidence interval for the area under the curve (AUC) was constructed. Data analysis was performed using the programming language and statistical software R (version 3.6.1).

## Results

The total number of patients included was 56. Of this total, 16 (28.6%) were female, with a mean age of 42.1 (range 18–73) years and a SD of 16 years. The mean diameter of the optic nerve for the total number of patients studied was 5.4 mm (range 3.8–7.3) with a standard deviation (SD) of 0.7 mm. The pathologies included in the sample were traumatic brain injury (69.6%), subarachnoid hemorrhage (26.7%), and intracerebral hemorrhage (3.6%) (Table 1). For ICP, the mean pressure for the total number of patients was 17.9 mmHg with a standard deviation of 7.7 mmHg and a range of 3.0 mmHg to 48.0 mmHg. If a cut-off point for ICP of 20 mmHg is considered, 14 (25%) patients presented with values higher than this measurement.

**Table 1:** Characteristics of the sample

	Mean ± SD	Range (min–max)
Age (years)	42 ± 16	55 (73–18)
ONSD	5.4 ± 0.7	3.5 (7.3–3.8)
	*n* = 56 (%)	
Sex		
Male	40 (71.4)	
Female	16 (28.6)	
Diagnosis		
TBI	39 (69.6)	
SAH	15 (26.7)	
ICH	2 (3.6)	

TBI, traumatic brain injury; SAH, subarachnoid hemorrhage; ICH, intracerebral hemorrhage

In our study, a logistic regression model was carried out in which it was observed that the ONSD variable is statistically significant in relation to ICP with a *p* value of 0.00803 (< 0.05).

**Fig. 3:**
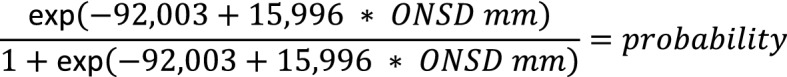
Logistic regression model (exp = exponential; probability = probability of finding elevated ICP)

This model estimates and predicts the probability that a patient will have an ICP greater than 20 mmHg (Fig. 3).

$$\frac{{\exp \left( { - \,92,003 + 15,996 \times {\text{ONSD mm}}} \right)}}{{1 + \exp \left( { - \,92,003 + 15,996 \times {\text{ONSD mm}}} \right)}} = {\text{probability}}{.}$$

From the model found, it is possible to estimate the probability of ICP > 20 mmHg from a given ONSD. For example, an individual with a diameter of 5.9 will have a probability of ICP > 20 mmHg of 0.9146 (Table 2).

**Table 2:** The different probabilities of presenting intracranial hypertension according to the ONSD measurement are observed

Cut-off point	Probability
5.5	0.0175
5.6	0.0813
5.7	0.3045
5.8	0.6843
5.9	0.9148
6.0	0.9815

For values greater than 5.7 mm, the probability of presenting intracranial hypertension increases considerably

**Fig. 4:**
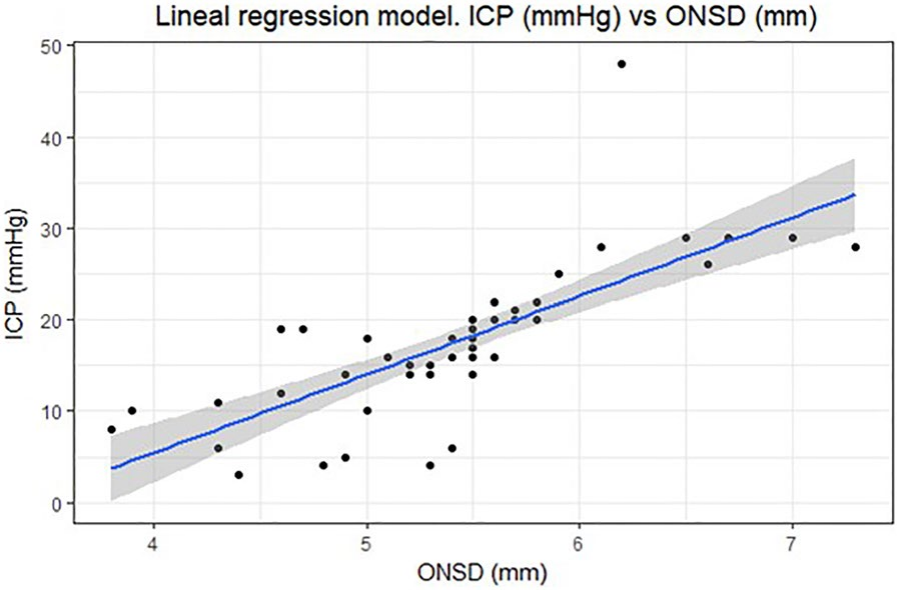
Linear regression model. In the analysis of the model, it is observed that the relationship between ONSD and ICP did not reflect a linear relationship for values greater than 6 mm of ONSD

To estimate the change in ICP, a lineal regression model was performed for each increase of 1 mm in the ONSD (Fig. 4), taking the ICP as a continuous variable measured in mmHg. It was observed that for each increase of 1 mm of ONSD there was an average increase of 8553 mmHg in ICP (Table 3). The explained variability by the model is 56.5%.

**Table 3:** For each increase of 1 mm of ONSD there was an average increase of 8553 mmHg in ICP

	Est	SE	*t* val.	*p* val.
(Intercept)	− 28.739	5.605	− 5.127	0.000
ONSD	8.553	1.021	8.375	0.000

Est., estimated effect; SE, standard error; *t* val., t value; *p* val., *p* value

In the analysis of the model, it is observed that the relationship between ONSD and ICP did not reflect a linear relationship for values greater than 6 mm of ONSD. The relationship between ICP and ONSD in this section can be studied by obtaining a larger amount of data.

**Fig. 5:**
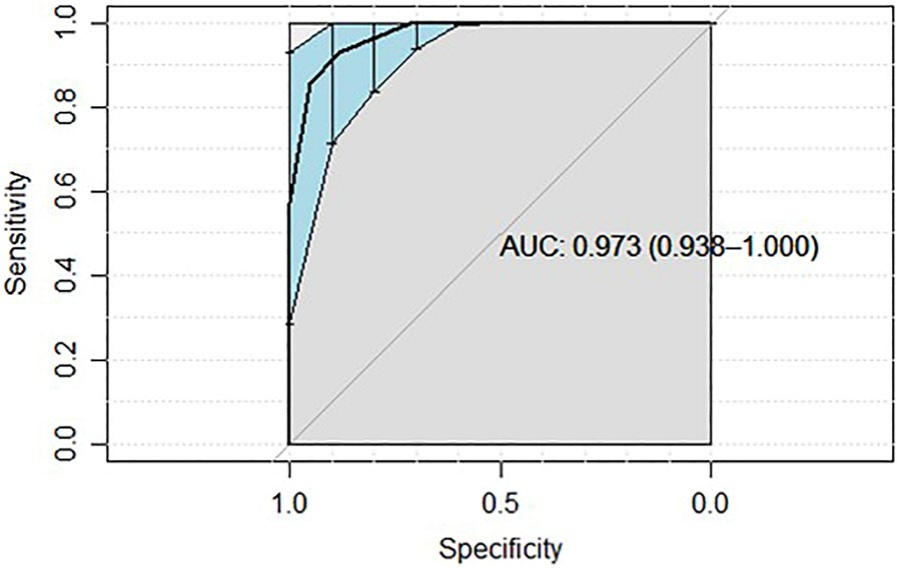
ROC curve. The area under the ROC curve (AUC) was 0.973. From the analysis of the cut-off points, it is observed that a value of 5.7 mm of ONSD maximizes the sensitivity (92.9%) of the method (a greater number of individuals with ICP > 20 mmHg are correctly identified) with a specificity of 88.1%

To find the cut-off point for ONSD that maximizes sensitivity to identify individuals with ICP > 20 mmHg, a ROC curve was constructed (Fig. 5).

The area under the ROC curve (AUC) was 0.973, with a 95% confidence interval of [0.938–1]. From the analysis of the cut-off points, it is observed that a value of 5.7 mm of ONSD maximizes the sensitivity (92.9%) of the method (a greater number of individuals with ICP > 20 mmHg are correctly identified) with a specificity of 88.1%.

## Discussion

Our study demonstrates that ONSD measured by ultrasonography is strongly related to ICP. The measurement of distension of the sheath that surrounds the optic nerve could be used to detect elevated ICP above 20 mmHg in neurocritical patients.

These results are in agreement with the findings of other researchers [10, 14, 19].

The present study incorporates a regression model from which the probability of finding elevated ICP from the ONSD measurement can be determined. This complements the binary decision (present/absent), which establishes a fixed threshold, and allows variation by considering different cut-off points for decision making.

Using a ROC curve, we have found a cut-off point of 5.7 mm ONSD to detect patients with ICP > 20 mmHg, which coincides with the results of other studies. However, we think that this method alone is insufficient. That is why, through our logistic regression model, it is possible to associate a probability of an ICP greater than 20 mmHg for each of the measured values of ONSD. For example, using the ROC curve, an ONSD of 5.7 mm would indicate an ICP greater than 20 mmHg. However, using the model, the same ONSD would indicate that there is a 30% chance that the ICP is greater than 20 mmHg.

To date, all studies have been based solely on finding a cut-off point for the ONSD to detect patients with ICP > 20 mmHg. None have included a model that maximizes the detection of these patients.

A meta-analysis by Dubourg et al. [20], which included six studies, shows a good level of diagnostic accuracy to detect intracranial hypertension in adult patients with traumatic brain injury and intracranial hemorrhage. Two of the studies included in this meta-analysis established a cut-off point of 5.2 mm, and in the remaining four studies, the cut-off points were 5.9, 5.0, 5.7, and 5.8 mm.

The meta-analysis by Chiara Robba et al. published in 2018 [21], which included seven studies, concludes that the ultrasound measurement of the ONSD can be a potentially useful technique to evaluate intracranial hypertension in binary mode (present/absent) when invasive monitoring methods are not desirable or are not available. However, due to the limited number of patients and the low quality of the studies included in this meta-analysis, larger high-quality studies are needed to establish the appropriate cut-off value for intracranial hypertension and the applicability of this technique in specific clinical conditions. In this meta-analysis, the ONSD threshold values that optimized the sensitivities and specificities evaluated by the ROC curves ranged from 4.80 to 6.30 mm.

Our working group for this study strongly supports the need for invasive ICP measurement in neurocritical patients with suspected elevated ICP, as well as all existing guidelines recommended for the measurement and monitoring of absolute ICP values. However, having a non-invasive estimate of the risk of ICP elevation is of interest when invasive ICP measurement is not available, is contraindicated, or there are doubts about its effectiveness given the patient's circumstances. The ultrasonographic measurement of ONSD to detect intracranial hypertension can be complemented by other non-invasive monitoring tools, such as transcranial Doppler. This ultrasound-based method is quick, low cost, and based on technology widely available in emergency departments and intensive care units [22, 23].

## Limitations

One limitation of our study is the low number of patients included (*n* = 56). However, it is important to highlight that we have performed only one measurement on a single day for each patient, which we see as a strength of the study since it complies with the principle of statistical independence. Lack of ultrasound experience is also an obvious limitation to the use of ocular ultrasound despite prior training in the technique.

While there were two operators performing the ultrasound measurements, an assessment of inter-operator variability was not conducted.

Two different methods of ICP monitoring were used (subdural and ventricular), which are not perfectly homogeneous.

Lastly, our study lacks a power analysis, so there is no sample size calculation.

## Conclusion

In this research work we set out to find the most appropriate cut-off point for the measurement of ONSD by ultrasound to diagnose intracranial hypertension. Since a single cut-off is insufficient for the diagnosis of intracranial hypertension, we have incorporated a logistic regression model from which the probability of finding elevated ICP from the ONSD measurement can be determined.

Through the following study, it is concluded that in sedated neurocritical patients, with structural acute brain injury, the measurement of the ONSD correlates with the ICP values measured invasively.

It was observed that with ONSD values less than 5.7 mm, the probability of being in the presence of ICP above 20 mmHg is very low, while for ONSD values greater than 5.7 mm, said probability clearly increases.

## Data Availability

The datasets used and analyzed during the current study are available from the corresponding author on reasonable request.
